# The experience of discrimination of individuals living with chronic hepatitis B in four provinces of China

**DOI:** 10.1371/journal.pone.0195455

**Published:** 2018-04-12

**Authors:** Bingfeng Han, Qianli Yuan, Yuhui Shi, Lai Wei, Jinlin Hou, Jia Shang, Ying Han, Cunduo Jin, Po-Lin Chan, Hui Zhuang, Jie Li, Fuqiang Cui

**Affiliations:** 1 Department of Laboratorial Science and Technology, School of Public Health, Peking University, Beijing, China; 2 National Immunization Program, Chinese Center for Disease Control and Prevention, Beijing, China; 3 Hepatology Unit, Peking University People’s Hospital, Beijing, China; 4 Hepatology Unit, Nanfang Hospital, Guangzhou, China; 5 Hepatology Unit, Henan Provincial People’s Hospital, Zhengzhou, China; 6 Hepatology Unit, Xijing Hospital, The Fourth Military Medical University, Xi’an, China; 7 Central sterile supply Department, 302 Military Hospital of China, Beijing, China; 8 World Health Organization China, Beijing, China; 9 Department of Microbiology and Center of Infectious Diseases, School of Basic Medical Sciences, Peking University Health Science Center, Beijing, China; Centre de Recherche en Cancerologie de Lyon, FRANCE

## Abstract

**Objectives:**

To assess chronic hepatitis B (CHB) patients’ knowledge about hepatitis B and their experience of discrimination with regard to study, work, and daily life.

**Methods:**

We administered a questionnaire to 797 CHB patients in four provinces of China and used one-way analysis of variance (ANOVA) and a generalized linear model (GLM) to identify factors associated with discrimination.

**Results:**

CHB patients had low levels of knowledge about hepatitis B. Patients under 40 years of age with a junior college education or above knew more about hepatitis B than CHB patients over 40 years of age who had only a high school education. Three-fourths of patients had experienced discrimination because of their hepatitis B infection, with no differences in the proportion experiencing discrimination by sex or age. People with more education reported less discrimination. Patients in Beijing and Henan province perceived less discrimination than those in Shaanxi and Guangdong provinces. Discrimination was significantly associated with negative emotions. CHB patients had little awareness of China’s anti-discrimination laws and policies. Among patients who had experienced discrimination, fewer than 10% knew organizations or institutions that could offer help. Over 60% of CHB patients who experienced discrimination chose not to respond.

**Conclusion:**

CHB patients in China commonly experienced discrimination, which was associated with significant, negative emotional stress. To mitigate the damaging effects of discrimination, our study suggests raising general population knowledge about hepatitis B, raising awareness of the availability of legal protection and organizations that can fight discrimination, and providing psychological support for CHB patients.

## Introduction

Hepatitis B virus (HBV) infection is an important public health problem globally, and in China. It is estimated there are 280 million individuals living with chronic hepatitis B worldwide and 90 million in China [1,2]. Due to misinformation about hepatitis B transmission, prevention, diagnosis, and treatment, and excessive fear caused by false advertisements that exaggerate the dangers of hepatitis B and its contagiousness, chronic hepatitis B (CHB) patients are known to experience discrimination. Discrimination implies being treated differently and unreasonably, often stigmatizing the individual [3–5]. There are HBV anti-discrimination laws and regulations in China, but despite these laws, discrimination is commonly seen in employment, education, health care, and daily life [3,4,6–10].

CHB patients suffer not only physical effects of their illness but also psychological stress that may be caused by discrimination. Studies have shown that HBV discrimination negatively impacts CHB patients’ feelings, and leads to fear, despair, anxiety, guilt, and avoidance behavior [4,10,11,12]. Avoidance of detection of one’s HBV status delays counseling and treatment and can negatively impact health outcomes while perpetuating transmission of HBV. Given the large number of people living with HBV infection in China, reduction of discrimination is critically important. There is, therefore, a need to assess the experience of discrimination status faced by CHB patients, but few studies of discrimination among Chinese CHB patients have been published. Studies of HBV discrimination have been conducted among college students [13,14], adults living in rural areas [4,8,15] and immigrants [11,16]. We report results of a study to assess quantitatively the experience of discrimination situation among CHB patients to identify factors associated with discrimination, with the goal of developing strategies to reduce HBV discrimination in China.

## Materials and methods

### Study sites and population

Based in part on the prevalence of hepatitis B surface antigen (HBsAg), the serological marker for CHB infection, we selected Beijing, Henan, Guangdong, and Shaanxi as study provinces. Although they cannot be representative of all of China, these four provinces provide variation in socioeconomic status and background rate of CHB. We selected a total of 5 hospitals (1 or 2 from each province) from which to sample CHB patients.

We invited CHB patients from liver disease clinics of the hospitals to participate in the study. Inclusion criteria included (1) at least 6 months of HBsAg positivity, (2) an alanine aminotransferase (ALT) level >40 IU/L, and (3) having physical symptoms or signs of hepatitis. CHB patients meeting the 3 inclusion criteria and who provided written consent to participate became the subjects of our study.

### Ethical review

The study was approved by the Peking University Health Science Center Ethical Review Board. Written, informed consent was obtained from participants. Personal identifying information was not retained in the analytic data sets.

### Survey tools and methods

The instrument that we used for measuring the experience of HBV discrimination was based on an instrument for assessing discrimination experienced by HIV/AIDs patients. This instrument was developed by AIDS/STD Division of Policy & Sociology in the Joint United Nations Program on HIV/AIDS (UNAIDS). The survey instrument was modified for our study after evaluation by hepatitis experts and pilot testing. Information collected included demographics (gender, age, education, and occupation); HBV-related knowledge; and subjects’ perceived experience of discrimination in medical treatment, employment, school, and reproductive health.

### Definitions

#### Hepatitis B related knowledge

This section had a total of 16 questions, covering how HBV is transmitted, diagnosed, and treated, and whether HBV infection can be prevented. One point was given for each correct answer; zero points were given for an incorrect answer; the possible score ranged from 0 to 16 points.

#### Negative emotions

Negative emotions (anxiety, depression, low self-esteem, loneliness, and sensitivity) were measured with 24 questions in a section called "inner feelings and behavior." Each item was scored 1 (yes) or 0 (no). The total possible score ranged from 0 to 24 points, and higher scores indicated more negative emotions and mental stress.

#### Hepatitis B discrimination index

The experience of discrimination was measured as a "discrimination index," which included both subjective and objective items. Subjective discrimination was defined as attitudes and reactions from others felt by CHB patients themselves after their infection status was revealed. Reactions of other people were classified as "no discrimination," "little discrimination," and "serious discrimination," and were assigned "0," "1," and "2" points, respectively. Possible scores ranged from 0 to 20 points, with higher scored indicating greater perception of discrimination.

Objective discrimination was defined as a personal experience of discrimination. For objective discrimination, there were 27 items, each describing a discriminatory experience; items were scored “1 point” (have experienced) or “0 point” (have not experienced). The possible score ranged from 0 to 27 points with higher scores indicating experiencing more discriminatory events.

The overall discrimination index was the unweighted sum of the subjective discrimination index and the objective discrimination index; it ranged from 0 to 47 points. The higher the discriminatory index, the more serious the perception and experience of discrimination.

### Data collection and statistical analysis

Epidemiology Data 3.1 was used for data entry by trained data management professionals. Statistical Product and Service Solutions (SPSS, version 22.0) was used for statistical analyses. Analytical methods included descriptive statistics, one-way analysis of variance (ANOVA), and a generalized linear model (GLM).

## Results

### Demographic characteristics

In total, 900 patients were invited to participate; 797 valid questionnaires were obtained, for a response rate of 88.6%. The male to female ratio was 2:1; subjects’ ages ranged from 18–77 years, with a median of 38 years; educational attainment was predominantly junior middle school / special secondary school / high school (44.2%), followed by bachelor degree (28.3%). The most common occupation was worker/peasant (26.7%), followed by “other” occupations (19.8%) and corporate staff (18.5%). (Table 1)

**Table 1:** Characteristics of subjects.

Demographic variable		Number	Effective denominator	Percentage (%)
**Gender**	Male	510	766	66.6
	Female	256	766	33.4
**Age**	≤30	229	787	29.1
	30–40	211	787	26.8
	41–50	181	787	23.0
	≥51	166	787	21.1
**Education**	Primary school and below	67	760	8.8
	Junior middle/special Secondary/high school education	336	760	44.2
	Junior college	142	760	18.7
	Bachelor degree or above	215	760	28.3
**Occupation**	Worker or peasant	208	780	26.7
	Teacher/researcher/medical Staff/civil servant	83	780	10.6
	Business/service/foreign/private Enterprise staff	144	780	18.5
	Self-employed persons	109	780	14.0
	Unemployed	81	780	10.4
	Other^a^	155	780	19.8

Note: The effective denominator refers to the number of respondents to the corresponding questions, excluding missing.

^a^ Including drivers, students, retirees and others.

### HBV knowledge

We used subjects’ knowledge scores as a dependent variable in one-way ANOVA, and used age, gender, education level, and province of residence as independent variables. There were no differences in knowledge scores by gender (F = 1.598, P = 0.207), but there were significant differences by age (F = 13.703, P<0.001), educational attainment (F = 46.815, P<0.001), and province (F = 5.867, P = 0.001). The Bonferroni method was used to conduct pairwise comparisons. We found that the mean knowledge score of patients under 40 years was significantly higher than that of patients over 40 years of age; the mean knowledge score of patients who graduated from junior college and patients having bachelor degrees or above was significantly higher than that of patients with high school education and below. The mean knowledge scores of subjects in Henan and Guangdong provinces were significantly higher than those of patients in Beijing and Shaanxi provinces. (Table 2)

**Table 2:** Hepatitis B knowledge by subject characteristics.

Demographic variable		Number	Knowledge score ($\bar{X}\pm S$)	F value	P value
**Gender**	Male	507	11.62±3.28	1.598	0.207
	Female	255	11.29±3.42		
**Age**	≤30^a^	227	12.22±3.20	13.703	<0.001
	30-40^a^	210	12.09±2.74		
	41-50^b^	181	10.83±3.46		
	≥51^b^	164	10.51±3.65		
**Education**	Primary school and below^a^	67	8.27±3.99	46.815	<0.001
	Junior middle/special Secondary/high school education^b^	335	10.87±3.19		
	Junior college^c^	140	12.44±2.64		
	Bachelor degree or above^c^	214	12.84±2.87		
**Province**	Beijing^a^	197	11.45±3.32	5.867	0.001
	Henan^b^	197	12.12±2.99		
	Guangdong^b^	198	11.63±3.39		
	Shaanxi^a^	200	10.75±3.54		

Note: Superscripted letters indicate that the difference between two items is statistically significant unless the letters are the same.

### Discrimination and its associated factors

More than three-fourths (511/677) of patients perceived or experienced discrimination by family, friends, colleagues, leaders, or healthcare workers because of their CHB infection. We developed a GLM, in which the dependent variable was the discrimination index and independent variables were age, gender, education attainment, province of residence, having negative emotions, and the hepatitis B knowledge score. There were no statistically significant differences in the discrimination index by gender and age grouping, but there were statistically significant differences between groups by education and region. The discrimination index of patients with a special secondary/high school education was significantly higher than that of patients with bachelor degrees or above. The discrimination index of patients in Shaanxi and Guangdong provinces was significantly higher than that of patients in Henan Province. The discrimination index of patients in Beijing was in the middle of the four provinces, but the difference was not statistically significant. There was a significant positive correlation between the discrimination index of patients and their negative emotions, but there was no statistically significant difference between the discrimination index of CHB patients and their knowledge score. (Table 3)

**Table 3:** Results of the generalized linear model of discrimination status and associated factors.

Potentially associated factor		Number	Discrimination index ($\bar{X}\pm S$)	Β value	t value	P value
**Gender**	Male	417	4.67±0.30	0.335	0.738	0.461
	Female^ref^	204	4.34±0.39			
**Age**	≤30	180	4.19±0.45	0.180	0.263	0.793
	30–40	166	5.10±0.45	1.087	1.637	0.102
	41–50	134	4.71±0.49	0.700	1.094	0.275
	≥51^ref^	141	4.01±0.50			
**Education**	Primary school and below	61	5.12±0.73	1.472	1.648	0.100
	Junior middle/special secondary/high school education*	270	4.84±0.34	1.194	2.160	0.031
	Junior college	109	4.41±0.52	0.755	1.180	0.238
	Bachelor degree or above^ref^	181	3.65±0.43			
**Province**	Beijing	148	3.80±0.48	-1.066	-1.771	0.077
	Henan*	166	3.54±0.45	-1.323	-2.187	0.029
	Guangdong	120	5.82±0.52	0.953	1.456	0.146
	Shaanxi^ref^	187	4.86±0.42			
**Negative emotions** *				0.538	12.059	<0.001
**Knowledge score**				-0.067	-0.958	0.339

* indicates p<0.05

^ref^ reference group

CHB patients also perceived or experienced discrimination in medical and reproductive health. Of respondents who were married or had children, 4.1% (28/675) had been advised not to have children; 4.8% (10/210) of female patients had been asked to terminate a pregnancy by medical staff or family planning department staff; 3.3% (22/644) and 3.2% (21/644) of CHB patients had been refused family planning services and reproductive health services, respectively.

### Legal protection or recourse

**Fig 1:**
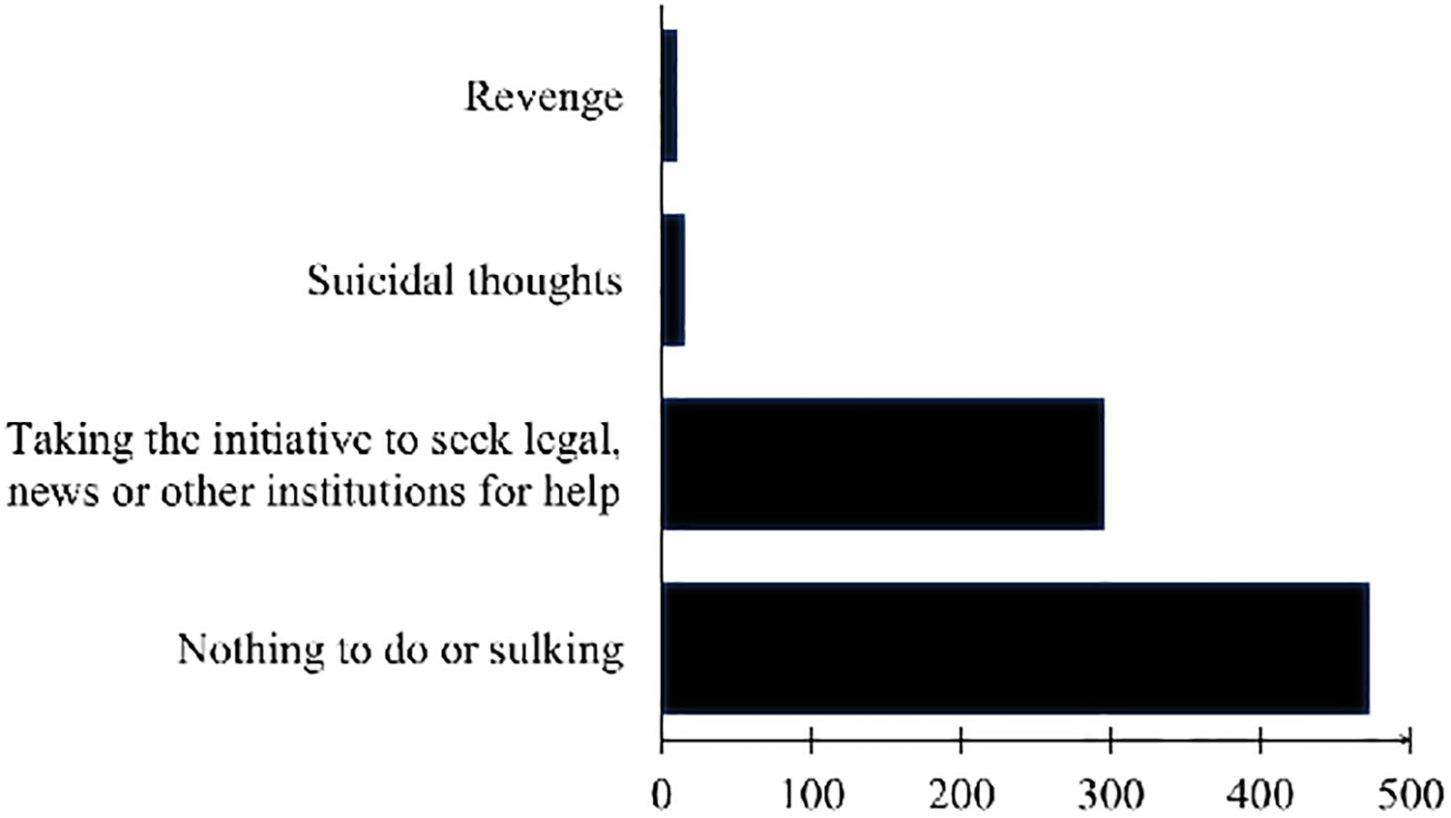
Choice of coping style if patients were fired because of hepatitis B infection.

China has laws and regulations intended to prevent or mitigate HBV discrimination. More than 40% (323/751) of subjects did not know that the《Notice on Cancellation of Hepatitis B Test Items in School Admission and Employment Examination》had been issued by the Ministry of Human Resources and Social Security (MOHRSS) of the People’s Republic of China in 2010. Approximately 60% (454/751, 449/751) of patients did not know that the《Civil Service Recruitment General Standard (Trial)》had been promulgated in 2005 and that the《Employment Service and Employment Management Regulations》had been promulgated in 2008, respectively. Fewer than 10% (73/772) of patients were aware of organizations or institutions that provide help to mitigate discrimination. If fired by a company because of HBV infection, more than 60% (472/767) of subjects chose to do nothing but keep silent; 3.26% (25/767) patients indicated they would “take revenge or commit suicide.” Fewer than 40% (296/767) of patients would seek legal help. (Fig 1)

## Discussion

Our study showed that three-fourths of Chinese CHB patients experienced discrimination because of their hepatitis B infection, and that many suffered severe discrimination [7, 17–19]. The experience of discrimination was associated with strongly negative feelings and mental stress. Only a minority of CHB patients knew about legal protections, legal recourse, and organizations that help fight HBV discrimination.

We also showed that CHB patients’ knowledge about HBV was low, especially regarding how HBV is transmitted—a finding that is consistent with other studies conducted in China and abroad [20–24]. Younger patients with more education had better knowledge about hepatitis B than those above 40 years old with less than a high school education. Given the significance of CHB, we think it is important to enhance education about HBV among CHB patients, especially older and less educated patients [25,26].

We found that the perception or experience of discrimination was unrelated to knowledge about HBV, a finding that is similar to a result from a study conducted in Beijing [9]. All of our subjects were CHB patients, which may partially explain the lack of association between knowledge and discrimination. An association between knowledge and discrimination can be seen in studies that include CHB patients and HBV-free individuals [4,9].

We found no association between discrimination and gender or age, but higher education was associated with less experience of discrimination. CHB patients with less than a college education experienced more discrimination than those with bachelor degrees or above. Because this study is based on a cross-sectional survey, cause and effect between educational attainment and discrimination cannot be determined. It may very well be that discriminatory events kept a CHB patient from advancing his or her education while at a younger age, or it could be that a CHB individual with higher socioeconomic status, for reasons of their status, may experience fewer discriminatory events.

CHB patients in Shaanxi and Guangdong provinces experienced more discriminatory events than patients in Beijing and Henan Provinces. Although our study is not able to determine why that is the case, a possible reason is that residents of four cities have different attitudes toward hepatitis B. Such a hypothesis can be tested with a study that evaluates community attitudes towards CHB patients.

Our finding that the experience of discrimination is strongly associated with negative emotions provides a strong rationale to identify methods to mitigate discrimination of CHB patients. Negative emotions can stem from the experience of discriminatory events, and can affect the perception of discrimination, causing a strongly positive correlation between negative emotions and discriminatory experiences [10]. Improving the psychological status of CHB patients, with psychological support and other interventions, should be part of anti-discriminatory efforts [27,28].

CHB patients also experienced discrimination in medical institutions and from healthcare workers. Of course, CHB patients can get married and give birth to a healthy baby by prevention of mother to child transmission of HBV [29,30]. Indeed, building on the success of 3 decades of work to prevent perinatal transmission of HBV [31], the government of China initiated an Integrated Prevention of Mother to Child Transmission (iPMTCT) program in 2010 that became a national program in 2015. The iPMTCT program screens all pregnant women for HBV markers and ensures the timely birth dose of HBV vaccine and initiates post-exposure prophylaxis of HBV-exposed newborn infants [32]. Findings in our study support the need for strengthening education and training of healthcare workers so that they can provide appropriate guidance and advice for all CHB patients.

We found that patients had limited awareness of their rights when facing discrimination, even though several anti-discrimination laws exist to guarantee legal rights of CHB patients. These laws include 《Civil Service Recruitment General Standard (Trial)》, promulgated in 2005,《Employment Service and Employment Management Regulations》, issued in 2008, and《Notice on Cancellation of Hepatitis B Test Items in School Admission and Employment Examination》, published by MOHRSS in 2010. We showed that few CHB patients were aware of these laws and policies. After experiencing a discriminatory event, less than 10% of patients turned to support organizations for help, even though anti-HBV-discrimination support organizations exist in China; most CHB patients remained silent after experiencing discrimination. Thus, there is an urgent need to promote awareness of legal recourse and supportive organizations among all CHB patients, but especially among those with low educational attainment.

There are some limitations in this study that should be considered when interpreting the results. First, the four provinces were selected by convenience sampling and cannot be generalized to the entire country. Second, although our questionnaire was based on another discrimination assessment instrument, this was the first use of our instrument, making comparison with other studies not yet possible. Third, our study was observational, and so only associations, but not causality, can be determined.

## Conclusion

There are large number of CHB patients in China, and the vast majority of these patients experience discrimination because of their CHB status. Strong negative emotions, depression, and stress are prevalent among CHB patients. Discrimination can have an impact on the response to hepatitis B in China, as fear of discrimination prevents individuals from determining their HBV status. Promoting awareness of legal protections and remedies, raising hepatitis B knowledge, and offering psychological intervention for CHB patients should be considered to protect patients from the harms of discrimination. Reducing discrimination can contribute to the development of social fairness in a more just and harmonious society.

## Supporting information

S1 File Shared database & meaning of variable name. (RAR)
